# Impact of a smartphone application (KAIA COPD app) in combination with *A*ctivity *M*onitoring as a maintenance pr*O*gram following *PU*lmonary *R*ehabilitation in COPD: the protocol for the AMOPUR Study, an international, multicenter, parallel group, randomized, controlled study

**DOI:** 10.1186/s13063-020-04538-1

**Published:** 2020-07-11

**Authors:** Marc Spielmanns, Tobias Boeselt, Stephan Huber, Pawandeep Kaur Bollinger, Bernhard Ulm, Anna-Maria Peckaka-Egli, Inga Jarosch, Tessa Schneeberger, Sabine Schoendorf, Rainer Gloeckl, A. Rembert Koczulla

**Affiliations:** 1 Pneumologie, Zürcher RehaZentren Klinik Wald, Faltigbergstrasse 7, 8636 Wald, Switzerland; 2 Faculty of Health, Department of Pulmonary Medicine, University of Witten/Herdecke, 58448 Witten, Germany; 3 Department of Pulmonary Rehabilitation, Philipps-University of Marburg, German Center for Lung Research (DZL), Marburg, Germany; 4 Fa. Kaia Health GmbH, Siegfriedstr.8, 80797 Munich, Germany; 5 Unabhängiges statistische Beratung Berhard Ulm, Kochelseestr 11, D-81371 Munich, Germany; 6 Institute for Pulmonary Rehabilitation Research, Schoen Klinik Berchtesgadener Land, Schoenau am Koenigssee, Germany; 7 Department of Prevention, Rehabilitation and Sports Medicine, Technical University of Munich (TUM), Munich, Germany

**Keywords:** Physical activity, Smartphone application, KAIA COPD, COPD, Digital therapeutics, Maintenance, Medical mobile application

## Abstract

**Background:**

Increasing physical activity (PA) is considered to be an important factor for the efficient management of chronic obstructive pulmonary disease (COPD). Successful methods required to achieve improvements in PA following pulmonary rehabilitation (PR), however, are rarely reported. Therefore, we will conduct this trial to evaluate the effectiveness of using a COPD management program delivered to the patient via the KAIA COPD app, a mobile medical application, after the completion of PR.

**Methods:**

This is the protocol for a randomized, controlled, open-label, multicentered trial that will be carried out at inpatient PR hospital centers in Germany and Switzerland. The interventions will involve the use of the KAIA COPD app program (Arm 1) or an active comparator, i.e., usual care (Arm 2). Patients completing an in-hospital PR program and consenting to participate in the study will be screened with the inclusion and exclusion criteria and enrolled in the study. After fulfilling the screening requirements, the patients will be randomized into one of the two arms with parallel group assignment in a 1:1 ratio. The training program will be delivered to the participants grouped in Arm 1 via the KAIA COPD app and to participants grouped in Arm 2 via the regular recommendations or standard of care by the PI. In total, 104 participants will be included in the trial. The treatment period will last for 24 weeks. Electronic versions of questionnaires will be used to collect patient-reported assessments remotely. The primary outcome measure is the change in physical activity of the intervention group in comparison to the control group, measured over 1 week as the mean steps per day with a Polar A 370 activity tracker, from baseline (end of PR) to the 6-month follow-up. The secondary outcome measures are functional exercise capacity, health status, sleep quality, exacerbation rate, and depression and anxiety symptoms assessed at several intervals.

**Discussion:**

This study seeks to prove the effects of the KAIA COPD mobile application in COPD patients after PR. The app offers educational, exercise training plus activity monitoring and motivational programs that can be easily implemented in the patient’s home setting, enabling patients to maintain the effects that are typically elicited in the short term after pulmonary rehabilitation for the long term.

**Trial registration:**

German Clinical Trials Register (DRKS00017275). Protocol version 2.0 dated 3 June 2019.

## Administrative information

**Table:** 

Title {1}	Impact of a smartphone application (KAIA COPD app) in combination with *A*ctivity *M*onitoring as a maintenance pr*O*gram following *PU*lmonary *R*ehabilitation in COPD: the protocol for the AMOPUR Study, an international, multicenter, parallel group, randomized, controlled study.
Trial registration {2a and 2b}.	German Clinical Trials Register (DRKS00017275).
Protocol version {3}	Protocol version 2.0 dated 17 December 2019.
Funding {4}	Clinical study sponsor: KAIA Health Software GmbH, Germany
Author details {5a}	Author details Spielmanns, Marc^1,2,8^; Boeselt, Tobias^3^; Huber, Stephan^4^; Kaur Bollinger, Pawandeep^4^; Ulm, Bernhard^7^; Pekacka-Egli, Anna Maria^1^; Jarosch, Inga^5^; Schneeberger, Tessa^5^; Schoendorf, Sabine^1^; Gloeckl Rainer^5,6^; Koczulla, A Rembert^3,5^ ^1,8^Pneumologie, Zürcher RehaZentren Klinik Wald, Faltigbergstrasse 7, 8636 Wald, Switzerland ^2^ Faculty of Health, Department Pulmonary Medicine, University of Witten/Herdecke, Germany ^3^ Department of Pulmonary Rehabilitation, Philipps-University of Marburg, German Center for Lung Research (DZL), Marburg, Germany ^4^Fa. KAIA Health GmbH, Siegfriedstr.8, 80,797 München, Germany ^5^ Institute for Pulmonary Rehabilitation Research, Schoen Klinik Berchtesgadener Land, Schoenau am Koenigssee, Germany ^6^ Department of Prevention, Rehabilitation and Sports Medicine, Technical University of Munich (TUM), Munich, Germany ^7^Unabhängiges statistische Beratung Berhard Ulm, Kochelseestr 11, D-81371 München ^8^corresponding author
Name and contact information for the trial sponsor {5b}	Dr. med. Stephan Huber, Chief Medical officer, Fa. KAIA Health GmbH, Siegfriedstr.8, 80803 München, Germany, Phone: +49 176 22355956, Email: stephan.huber@KAIAhealth.com Dr. Pawandeep Kaur Bollinger, Clinical Project Manager, Fa. KAIA Health GmbH, Siegfriedstr.8, 80803 München, Germany, Phone :+498920207057, Email : pawan.kaurbollinger@KAIAhealth.com
Role of sponsor {5c}	Study design, protocol writing, clinical data management, analysis, and interpretation of data; writing of the report; and the decision to submit the report for publication as per conditions laid out in the Sponsor-Investigator agreement

## Introduction

### Background and rationale {6a}

Exercise training is an important component in the management of COPD. Numerous trials have shown large improvements in health-related quality of life (HRQoL) and exercise capacity in subjects with COPD following structured exercise training within a pulmonary rehabilitation (PR) program [1]. However, despite the well-known benefits of exercise training, the great majority of patients who would benefit from PR are often not referred to such a program. The proportion of COPD patients who are not transferred to available pulmonary rehabilitation programs or who do not complete them is estimated to vary between 8 and 50% and between 10 and 32%, respectively [2]. Major barriers for not participating in or adhering to PR include travel and transport, disruption to an established routine, lack of perceived benefits, being socially isolated, and lack of social support, inconvenient timing, influence of health care provider, illness and comorbidities, current smoking, and depressive symptoms [3–5]. Additionally, for patients who undergo a supervised exercise training program, it is often challenging to implement a follow-up program or regular exercise training into their daily life home settings. Consequently, many COPD patients are either not instructed to exercise at all or fail to adhere to exercise training at home after completing PR [6].

Traditionally, PR programs are provided under direct supervision at a rehabilitation center with participants attending PR in an inpatient or outpatient setting. However, both community-based and home-based programs are becoming increasingly popular [6]. Of the studies included in the most recent systematic review of pulmonary rehabilitation in COPD patients [1], approximately 25% were home-based programs (*n* = 15) or included an element of home-based training along with an inpatient or outpatient program (*n* = 9). Many of the studies relied on supervision by medical staff in regular visits to a hospital or clinic for additional exercise training sessions, home-based visits, or telephone calls [7–13]. However, the strong effects detected immediately after completion of the exercise training programs either decreased or vanished in the long term after 12 months [14, 15].

These results in the abovementioned studies illustrate the potential of implementing digital additions to conventional PR. A recent British study investigated a smartphone-based intervention for PR. That study aimed to compare the clinical delivery of a 6-week online PR program (myPR) to the current clinical standard face-to-face PR delivered in a conventional community setting in patients with COPD. The authors found that the app was noninferior to the outpatient program and did not influence the occurrence of adverse events [16]. The KAIA COPD app is available as a digital solution for pulmonary rehabilitation in German-speaking countries. The COPD app is comprised of an exercise training program and an educational program that was developed by health care professionals and PR experts. In a feasibility study, promising effects from a 20-day Intervention with, this digitalized PR program on CAT and HRQoL in severely symptomatic COPD patients were found [17]. However, until now, there has been no evidence for the efficacy of the COPD app from prospective studies, especially following PR.

We will conduct this randomized controlled trial (RCT) to determine the effects of the KAIA COPD app versus usual care on PA in patients with COPD following an inpatient PR program.

Our aim is to test a smartphone application for COPD patients that presents an educational and exercise training program plus activity monitoring and a motivational program that can be easily implemented in the patient’s home setting and that may enable patients to maintain the effects that are typically elicited in the short term after pulmonary rehabilitation for the long term. The overall objective of this project is to assess the effectiveness of this smartphone application for COPD patients in the maintenance of physical activity following PR. Activity monitoring will be provided by an activity tracker (Polar A 370, Polar Elektro GmbH, Germany).

### Objectives {7}

The overall objective is to assess the effectiveness of the newly developed COPD app as a maintenance program after PR. The primary objective is to assess the clinical efficacy of the COPD app maintenance program on physical activity measured in steps/day in patients with COPD after 6 months. The secondary objectives are to evaluate the effects of the COPD app program on functional exercise capacity, health-related quality of life (HRQoL), patient-reported health status, exacerbations, and depression and anxiety symptoms. Furthermore, we aim to explore patient compliance/adherence and safety, identify factors that facilitate the implementation of the program in the patient’s home setting, and evaluate factors of the program that are especially supportive for the patients.

### Trial design {8}

This trial is an international, multicenter, parallel-group, randomized, controlled study. Study participants are stage II–IV COPD patients who will be randomly assigned to the intervention group that will be provided with the KAIA COPD app or to a control group that will receive usual care. In total, 104 participants will be randomized (52 participants will be allocated to the intervention group and 52 to the control group). Two centers specializing in inpatient PR for COPD (Schoen Klinik Berchtesgadener Land, Germany and Zuercher RehaZentren Klinik Wald, Schweiz) will recruit study participants.

The estimated duration of the main study phase is planned to last approximately 1.5 years. All potential participants are COPD patients who are taking part in a regular inpatient PR program. The study started with screening of the first participant in August 2019. Enrollment of participants in the study is planned for a 12-month period. The last patient to complete the program is expected to finish in February 2021.

## Methods: participants, interventions, and outcomes

### Study setting {9}

The study will take place at hospitals specializing in rehabilitation programs for COPD patients of all stages (I–IV gold stages). At present, 2 study sites are participating in the trial: Pneumologie, Zürcher RehaZentren Klinik Wald, Faltigbergstrasse 7, 8636 Wald, Switzerland, and the Institute for Pulmonary Rehabilitation Research, Schoen Klinik Berchtesgadener Land, Schoenau am Koenigssee, Germany.

### Eligibility criteria {10}

During the recruitment period, all patients with COPD admitted to the PR hospitals will be informed about the ongoing study. Patients who are interested in study participation will receive oral and written information about the study before signing the informed consent for. After signing the informed consent form, participants will be instructed on how to use the activity tracker (Polar A 370) by the local study personnel. Participants will be instructed to wear the activity tracker every day for at least 20 h per day. After the screening period (approximately 8–10 days), eligibility will be judged by the local investigators based on the randomization criteria. If the screening requirements are fulfilled and the participants are deemed eligible, they will be randomized.

Every 2 weeks, an e-link leading to the questionnaires will be sent via email regarding exacerbations (questionnaire defined by the GOLD Guidelines), safety (adverse events or severe adverse events), device deficiencies, and patient feelings. There will be no prohibited medications during the study, and any change in concomitant medications will be noted down biweekly. In addition, at baseline and after 3 and 6 months, another e-link will be sent via email to all participants to complete the CRQ and HADS, to provide instructions to perform the 1-min sit-to-stand test and COPD Assessment Test (CAT), and to evaluate whether participants met their personal goal of physical activity.

**Fig. 1:**
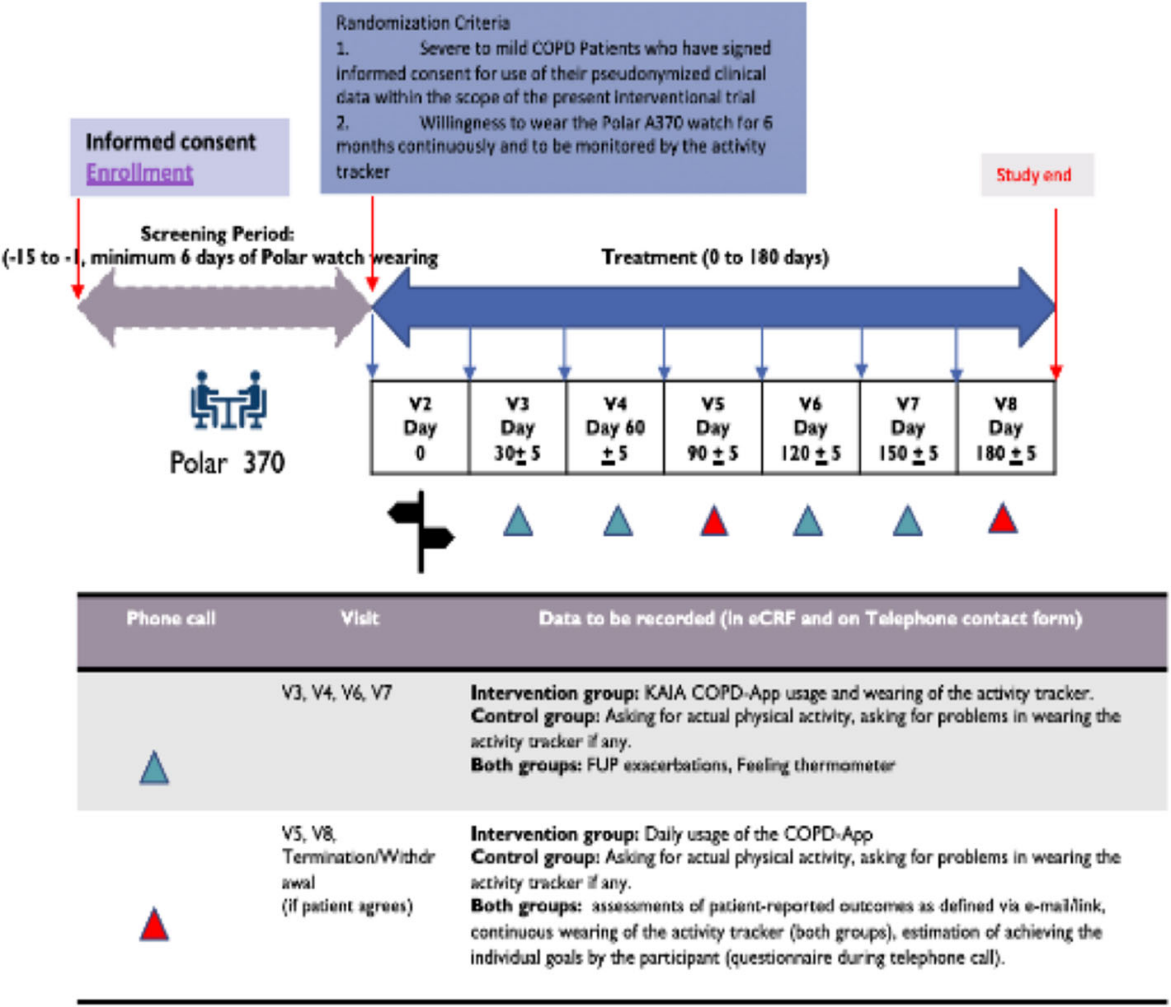
Provides an overview of the study conduct

Daily steps, sleep efficacy, and total sleep time will be passively collected by a Polar A 370 watch and will be imported to the study-specific electronic case report form (eCRF) system over the Google Fit Server. Figure 1 provides an overview of the study procedures.

The participants in the KAIA COPD app will be supplemented with a smartphone and access to the application together with the activity monitor. The participants in the control group will be supplemented with a smartphone and activity monitor. The smartphone is required in both groups to synchronize the daily steps from the activity monitor to the Polar website/database. These materials will be provided by KAIA Health Software GmbH for study purposes. At the end of the treatment period, the supplementary equipment will be collected from the participant.

#### Patient inclusion criteria

- COPD patients willing and able to sign the informed consent form for use of their pseudonymized clinical data within the scope of the present interventional trial
- COPD patients who have completed an in-hospital pulmonary rehabilitation program for an average duration of 3 weeks
- Diagnosis of COPD, defined as forced expiratory volume in 1 s/forced vital capacity (FEV1/FVC) < 70% predicted, FEV1< 80% predicted after bronchodilation, with or without chronic symptoms (cough, sputum production) corresponding to GOLD stage II–IV
- Completion of an inpatient pulmonary rehabilitation program
- Completion of the screening period and fulfillment of the randomization criteria as defined by the protocol
- Ability to use a smartphone and smartphone apps
- Willingness to wear an activity tracker during the 6-month study period
- Age ≥ 40 years of age
- Knowledge of German language to understand the study material, assessments, and contents of the COPD app

#### Patient exclusion criteria

- The patient is unable to conduct the exercise training program due to physical, cognitive, or safety reasons, as judged by the investigators, e.g., lower limb joint surgery within the preceding 3 months, unstable cardiac diseases, predominant neurological limitations, and planned surgical or other interventions disturbing the study intervention
- Significant psychiatric disorders, legal incapacity, or limited legal capacity. Patient participation in another clinical trial with an investigational medication within 30 days prior to study entry
- Patients already using the KAIA COPD app

#### Eligibility criteria during the screening period and before randomization

- Completion of the training program for home settings, supervised by the study nurse
- Daily almost full-time wearing of the Polar watch (at least 20 h a day)

#### Procedures after randomization

The study nurse will carry out a short training program (20 min) to demonstrate the use of the wearable and mobile device platform and smartphone for both groups.

### Who will take informed consent? {26a}

Patients will be provided with a written informed consent form by the PI or the study staff delegated to obtain informed consent, such as a subinvestigator. Eligible patients will receive written information about all relevant aspects of the study and that their participation in the study is voluntary and they have the right to refuse or withdraw their consent at any time without reprisals. The study has been registered with the German Clinical Trials Register (DRKS00017275).

This paper contains the original study protocol. Any substantial modifications to the study protocol will be submitted to the corresponding country Ethics Committee for approval prior to implementation. These amendments will be documented in detail in the German Clinical Trials Register and will be described transparently in trial reports.

### Additional consent provisions for collection and use of participant data and biological specimens {26b}

On the consent form, participants will be asked if they agree to the use of their data should they choose to withdraw from the trial. Participants will also be asked for permission for the research team to share relevant data with people from the universities taking part in the research or from regulatory authorities, where relevant. This trial does not involve collecting biological specimens for storage.

### Interventions

#### Explanation for the choice of comparators {6b}

The control group will also wear the activity tracker every day and use the smartphone for the assessments but have no access to the content of the COPD app. The control group will also be an active control as participants will receive a leaflet to encourage an active lifestyle (“Besser leben mit COPD”) and an individually composed list of exercises for home training from experienced physiotherapists at the end of PR. This procedure represents the recent standard of care in this field. After finishing the study, at the 6-month assessments (visit 8), participants who were allocated to the control group will be offered the KAIA COPD app for free.

#### Intervention description {11a}

The study intervention consists of training sessions conducted daily by the patient via the COPD app. Regular contacts via telephone calls will be conducted by the same health care professional with respect to the realization and execution of the exercise training program shown in the COPD app. Patients will be contacted by phone if they do not meet the exercise criteria (exercising with the app at least 4 out of 7 days a week).

#### Individualized strength training program

The exercise training program provided by the COPD app consists of a variety of whole-body exercises that will be performed on a daily basis for approximately 15–20 min. Simple sit-to-stand exercises to train large muscle groups in various forms and trunk and upper body resistance exercises that will be performed without supplemental equipment are major components of the training program. Daily strength training always begins with a whole-body warm-up and finishes with two to three appropriate stretching exercises. During the program, the intensity of exercises will progressively increase. All exercises will be explained in exercise videos, which include detailed instructions to ensure the correct performance, training amount, and training intensity. To document the training progress, the patients will record their completed training sessions in the COPD app, which will also be used to monitor training adherence by health care professionals.

#### Exercise concept

The general principle of physical exercise in the physiotherapeutic section of the app is to promote exercise with a focus on strength over endurance training. The exercises are chosen to affect several body parts at the same time. Exercise duration and intensity will initially be chosen depending on the user’s exercise capacity as assessed in the self-test in the app. Using constant reassessment, exercise intensity can be adapted after each day of use of the app. Overall, there are 47 exercises, mostly focusing on strengthening major muscle groups. Exercises vary from easy sitting exercises, for users with a high level of impairment, to standing or lying exercises. All exercises can be performed using a simple training mat, a chair, or sometimes a 1-l bottle of water, without the need for other items. Furthermore, the app promotes overall physical activity in terms of walking. The number of steps each day is collected by the wearable device and reported to the app, and goals for activity will be set weekly depending on the recent individual fitness level. Users are reminded of their activity goals via push notifications in the app.

The COPD app will be used by participants throughout the entire interventional period of 24 weeks.

#### Criteria for discontinuing or modifying allocated interventions {11b}

Each participant will be informed that participation in the study is voluntary, that he/she may withdraw from the study at any time, and that withdrawal of consent will not affect his/her subsequent medical assistance and treatment. The study might be prematurely terminated due to ethical concerns or insufficient participant recruitment or when the safety of the participants is doubtful or at risk, when alterations in accepted clinical practice make the continuation of a clinical trial unwise or when there is early evidence of benefit or harm of the experimental intervention.

For participants who prematurely stop the study, follow-up assessments that are normally conducted during visits 5 and 8 will be conducted at the point of termination only if the participants agree.

#### Strategies to improve adherence to the interventions {11c}

There will not be any additional strategies to improve adherence to the intervention.

#### Relevant concomitant care permitted or prohibited during the trial {11d}

Concomitant care will be allowed to continue during the study period.

### Provisions for posttrial care {30}

Provisions, if any, for ancillary and posttrial care and for compensation to those who suffer harm from trial participation will be provided.

### Outcomes {12}

During the study, no in-person visits will be planned for the study participants, and the assessments will be performed using electronic patient-reported outcomes (ePRO) by sending links to the participants. They will perform all required assessments by themselves, and the results will be directly transferred to the eCRF. The results of the assessments will be checked regularly by the study personnel of the site where the participants were recruited.

Daily steps, sleep efficacy and total sleep time measured passively and continuously for each participant by the activity tracker during the treatment period will be transferred and saved to the study eCRF database by Google Fit.

#### Assessment of the primary outcome

The primary outcome assessment will be the measurement of physical activity, recorded as steps every day (provided by the activity tracker Polar A 370), for 6 months for the intervention group as well as for the control group. Data regarding the steps per day will be transferred from the Polar watch to the smartphone via Bluetooth. Data from the KAIA COPD app will be sent to the KAIA database server via the internet. Therefore, all participants will be equipped with a smartphone that has internet access at home (the intervention group will also have access to the COPD app) and with a Polar watch (see Fig. 1).

#### Assessment of secondary outcomes

Secondary outcomes will be measured at three time points: during the baseline visit and at the 3- and 6-month follow-up visits/early termination visits via an email link sent by the study staff.

#### Assessment of explanatory outcomes

Explanatory outcomes are patient compliance with the training and the health professionals’ feedback. The satisfaction with the COPD app will be examined with 2 questions (English version, originally in German):

1. How satisfied are you with the course of your therapy after using the COPD app?
  - Very satisfied
  - Satisfied
  - Not satisfied
2. Would you recommend the KAIA COPD app to other patients?
  - Yes
  - No

Achievement of individual goals will be assessed by the participant himself with a questionnaire during a phone call.

Patient compliance and adherence to the training will be assessed every week by the study nurse, who will check the downloaded and viewed training videos (intervention group) and the daily activity, as captured by the activity tracker, of the participants (both groups). In addition, compliance will be assessed by the number of successfully conducted telephone calls (which will be reported by the health professionals who conduct the interventions).

Satisfaction and experience with the COPD app will be assessed by a satisfaction questionnaire at the 6-month follow-up visit (intervention group only).

### Participant timeline {13}

**Fig. 2:**
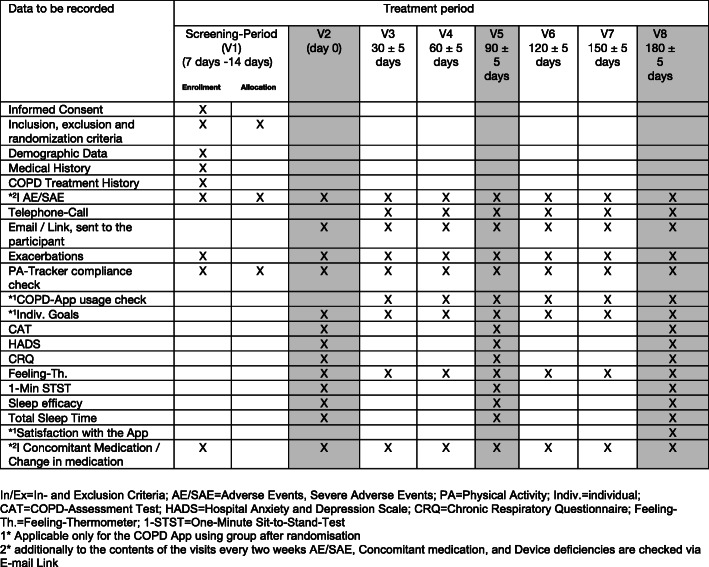
The study schedule in detail

The study will take approximately 1.5 years to completion. The screening period is scheduled after a minimum of 7 days and a maximum of 14 days during inpatient rehabilitation. Once the participant enters the study and is randomized, the treatment period lasts for 6 months with biweekly follow-up for SAEs/AEs and device deficiencies. Any participant who is not responding to the visit emails will be followed up with 3 times until being considered lost to follow up. Figure 2 displays the study schedule in detail.

### Sample size {14}

In total, 104 participants are expected to be recruited, 52 for the intervention group and 52 for the control group each. The sample size calculation is based on the primary outcome, the difference between groups in the average steps per day within 1 week at the 6-month follow-up. As this study had no pilot study, there are no further statistical sample size considerations. The well-established minimally important difference in steps per day after pulmonary rehabilitation lies between 600 and 1100 steps as measured with an activity tracker during an intervention. Assuming a standard deviation of the outcome variable of 2500 steps/day, an expected effect size of 2000 steps/day can be assumed for programs combining physical activity counseling with PR to increase activity, according to current meta-analyses. To achieve 80% power and a significance level of 5% (one-sided), a sample size of 26 patients in each group is required, which results in a total sample size of 104, assuming a drop-out rate of 50%. The sample calculation is based on a one-tailed nonpaired *t* test comparing the two groups with each other at the final visit at the end of the follow-up period.

### Recruitment {15}

The expected rate of recruitment from the centers is 2–3 patients per week. In case the trend does not follow with the theoretical recruitment rate, an additional site in either of the countries (Germany or Switzerland) could be considered.

Assignment of interventions: allocation

### Sequence generation {16a}

Randomization will be accomplished by using a software randomizer (https://www.randomizer.org).

### Concealment mechanism {16b}

For allocation concealment, the fabrication of lists will be done by the sponsor, and the allocation of each individual participant (using only ID, no personal data) will only be made available to investigators via email after reporting a patient being successfully included in the study to the sponsor. On the concluding day of the screening visit, if a patient is rendered eligible to participate in the study, the study staff will inform KAIA’s project management. After receiving the email, patients will be assigned to the corresponding group by the study center.

### Implementation {16c}

Randomization will be performed on the patient level with a 1:1 ratio using block randomization with varying block sizes, stratified per study center. Once the random number is delivered to the study site, it is the duty of the site personal to implement the procedures as specified in the protocol.

The study nurse will carry out a short training program (20 min) to demonstrate the use of the wearable and mobile device platform (smartphone) for both groups. Additionally, the following steps will be taken to explain the group assignment to the subjects.

#### Intervention group

- Explanation of the 3 dimensions of the COPD app (exercise, education, relaxation)
- Starting of the COPD app and a training session
- Setting the individual goals for physical activity (steps per day).
- Synchronization of the smartphone

#### Control group

- Setting the individual goals for physical activity (steps per day)
- Synchronization of the smartphone

### Assignment of interventions: open label study

#### Who will be blinded {17a}

Patients cannot be blinded due to the nature of the intervention. Blinding of statisticians or data managers will be carried out.

#### Procedure for unblinding if needed {17b}

The design is open label, so unblinding will not occur.

### Data collection and management

The investigators are responsible for ensuring the accuracy, completeness, and timelines of the reported data. All source documents will be completed in a neat, legible manner to ensure accurate interpretation of the data. Data reported in the eCRF derived from source data documents will be consistent with the source documents.

### Plans for assessment and collection of outcomes {18a}

Electronic data capture (EDC) will be used for this study. The study data will be transcribed by study-site personnel from the source documents into an electronic CRF and transmitted in a secure manner to the sponsor within the time agreed upon between the sponsor and the study site. The electronic file will be considered to be the eCRF. Olyro (www.olyro.de) will be used as the software to provide eCRFs and the database. eCRFs for each enrolled study participant will be filled in with all relevant data pertaining to the participant during the study.

Documented medical histories and narrative statements relative to the participants’ progress during the study will be maintained. These records will also include the following: originals or copies of laboratory and other medical test results (e.g., ECGs) which must be kept on file with the individual participant eCRFs. All data entered into the eCRF for the baseline visit, with the exception of clinical data, such as comorbidities, medication, and lung function measurements, which are taken from the electronic patient records in the clinics by the study nurses, will be source data and must also be available in the individual participant file either as print-outs or as notes taken by either the investigator or another responsible person assigned by the investigator.

Secondary outcomes (i.e., all patient-reported outcomes) will be reported by patients directly after sending a link to the patient that leads the patient directly to a webpage with the questionnaires mentioned above. Patient-reported outcomes will be collected via a web-based platform using individualized links sent to patients enrolled in the study and collected in the database. Personnel will not be given access to the system until they have been trained. Automatic validation procedures within the system check for data discrepancies during and after data entry and, by generating appropriate error messages, allow the data to be confirmed or corrected online by the designated investigator site staff. The investigator must certify that the data entered are complete and accurate.

Essential documents will be retained after the regular end or premature termination of the respective study according to the local regulatory requirements.

### Plans to promote participant retention and complete follow-up {18b}

For participants who withdraw consent, already collected data will be used for analyses, and if feasible and the participant agrees, the usual follow-up assessments will be conducted for data capture.

### Data management {19}

The data entry, coding, security, and storage procedures, including any related processes to promote data quality, will be performed according to the SOPs and the data management and validation plan provided by the sponsor and under the supervision of the designated clinical data manager of the sponsor.

### Confidentiality {27}

Only the study nurses and designated investigator site staff will enter the data required by the protocol into the system. Direct access to source documents will be permitted for purposes of monitoring, audits, and inspections.

### Plans for collection, laboratory evaluation, and storage of biological specimens for genetic or molecular analysis in this trial/future use {33}

This trial does not involve the collecting, laboratory evaluation or storage of biological specimens for genetic or molecular analysis.

Statistical methods

### Statistical methods for primary and secondary outcomes {20a}

#### Primary analysis

Baseline characteristics of the study participants will be summarized according to numbers and percentages for qualitative variables, means, and standard deviations for quantitative variables with normal distributions and medians and 25th–75th percentiles for quantitative variables with nonnormal distributions. Differences in the change in the primary outcome from baseline to the 6-month follow-up between the intervention and control group participants will be compared by linear regression analyses corrected for relevant confounders and baseline values. The same analysis will be used for continuous secondary outcomes. The primary analysis will be done with a one-sided nonpaired *t* test comparing the two groups with each other at the final visit at the end of the follow-up period.

For the main analyses, we will compare the control group with the intervention group using an intention-to-treat approach. This study is designed as a superiority study.

#### Per-protocol analysis

As a sensitivity analysis, we will additionally conduct a per-protocol analysis, where we will also compare the control group with the intervention group but where we will only retain those participants in the intervention group who adhered to the intervention for the analyses.

We define adherence to the protocol (in terms of exercise training) as conducting the exercise training sessions for at least 70% of the weeks the patients are able to train (i.e., weeks when patients suffered from exacerbations or other health conditions that prevent them from training will not be considered; as discussed with the coach), for at least 4 times a week, during the 6 months of the intervention (see the following definitions):

- Definition of a fulfilled individualized training session per day (according to the protocol: 3 different exercises per day are scheduled): completion of at least 2 different exercises per day
- Definition of a fulfilled week of training (rate of training sessions according to the protocol: 4 per week): completion of at least 4 out of the planned 7 individualized training sessions per week on average.

We will adjust the per-protocol analyses with prognostic factors for the primary outcome steps per day, lung function (FEV1), exercise capacity and exacerbation events, for adherence to conduction of the exercises (self-efficacy to conduct the exercises, exercise capacity, dyspnea, and acute worsening of health state [exacerbations and other]) and for whether the patients conducted other exercise strength training.

Concerning the control group, we will collect as much information as possible, especially with respect to the type and frequency of self-organized exercise training. This will also be included in the per-protocol analysis.

#### Secondary analyses

The explanatory outcomes are the patients’ compliance with the training, and their satisfaction with the exercise program will be analyzed using quantitative and qualitative methods, depending on the level of the data. All these analyses will be described in more detail in the statistical analysis plan in a separate document after the finalization of the protocol.

#### Safety analysis

All adverse and serious adverse events that are recorded during the study will be described with regards to their severity grade, relationship to the COPD app intervention, and duration. The occurrence of adverse events and AEs overall between groups will be assessed with a two-tailed *t* test after the end of the follow-up.

### Deviation(s) from the original statistical plan

Deviations from the intended statistical plan will be reported to the responsible ethics committee before implementing them, and potential amendments in the protocol will be made. They will furthermore be justified and listed in the final report.

### Handling of missing data and drop-outs

As described above, the main analyses will be conducted with an intention-to-treat approach, and missing follow-up data for patients who do not complete the V8 assessments will be substituted with baseline assessment data or the last known data (i.e., last outcome carried forward).

### Interim analyses {21b}

#### There are no anticipated problems that will be detrimental to the participant

##### Methods for additional analyses (e.g., subgroup analyses) {20b}

Additional analyses are planned according to COPD stage, age, comorbidities, and 6-MWT at discharge from PR.

##### Methods in the analysis to handle protocol nonadherence and any statistical methods to handle missing data {20c}

Missing follow-up data for patients who do not complete the V8 assessments will be substituted with the last available measurement (i.e., last outcome carried forward).

##### Plans to give access to the full protocol, participant level-data, and statistical code {31c}

The datasets analyzed during the current study will be available from the corresponding author on reasonable request.

### Oversight and monitoring

#### Composition of the coordinating center and trial steering committee {5d}

The principal investigator is responsible for all aspects of local organization, including identifying potential recruits and obtaining consent. She or he will also supervise the trial and how often the local investigator team meets. The Trial Steering Committee (TSC) is composed of the two principal investigators and the sponsor medical lead. They will meet over the course of the trial at least bimonthly to oversee the conduct and progress of the trial. There is not a plan to integrate a public reference group due to the design of the study.

#### Composition of the data monitoring committee and its role and reporting structure {21a}

A data monitoring committee will not be assembled; however, monitoring will be carried out by the assigned clinical study monitor from the sponsor. One regular monitoring visit organized by the sponsor will be planned at the investigator sites prior to the start and during the course of the study. All collected source data and other documents will be monitored.

During monitoring, the internal monitor will check the completeness of patient records, the accuracy of entries on the eCRFs, the adherence to the protocol regarding the assessments and Good Clinical Practice guidelines, and the progress of enrollment. Internal monitoring will be conducted according to a detailed monitoring plan.

### Adverse event reporting and harms {22}

Adverse events (AEs) are defined as any untoward medical occurrence in a patient or clinical investigation participant after the intervention which does not necessarily have a causal relationship with the treatment. An AE can therefore be any unfavorable and unintended sign (including an abnormal laboratory finding), symptom, or disease temporally associated with the intervention, whether or not related to the intervention. An AE may also consist of a new disease, an exacerbation of a pre-existing illness or condition, a recurrence of an intermittent illness or condition, a set of related signs or symptoms, or a single sign or symptom.

Serious adverse event

A serious adverse event is defined as any event that

- Requires inpatient treatment not envisaged in the protocol or extends a current hospital stay;
- Results in permanent or significant incapacity or disability;
- Is life-threatening or results in death; or
- Causes a congenital anomaly or birth defect.

All serious adverse events that occur after visit 1 will be collected in the eCRF. Medical conditions/diseases present before starting the study are only considered adverse events if they worsen after starting the study. Directly after registration, AEs and SAEs are judged by the PI to be related/not related or probably related to the intervention.

Adverse events occurring during the study will be recorded on the Adverse Events eCRF with the following information:

- The severity grade (mild, moderate, severe)
- The relationship to the KAIA COPD app intervention (suspected/not suspected)
- The duration (start and end dates)

Once an adverse event is detected, it will be followed until its resolution or until it is judged to be permanent, and an assessment will be made at each contact (or more frequently, if necessary) of any changes in severity, the suspected relationship to the intervention, the interventions required to treat it, and the outcome.

### Frequency and plans for auditing trial conduct {23}

A quality assurance audit/inspection of this study may be conducted by the CEC. The quality assurance auditor/inspector will have access to all medical records, the investigator’s study-related files and correspondence, and the informed consent documentation that is relevant to this clinical study.

The investigator will allow the persons responsible for the audit or inspection to have access to the source data/documents and to answer any questions arising. All involved parties will keep the patient data strictly confidential.

### Plans for communicating important protocol amendments to relevant parties (e.g., trial participants, ethical committees) {25}

This paper contains the original study protocol. Any substantial modifications to the study protocol will be submitted to the Ethics Committee for approval prior to implementation. These amendments will be documented in detail in the German Clinical Trials Register and will be described transparently in trial reports. An amendment affecting the risk and benefit statement will be given to the participants in a new version of the patient informed consent form.

### Dissemination plans {31a}

After the statistical analysis of this trial, the sponsor will make every endeavor to publish the data in a medical journal. The site investigators who contributed relevant scientific input will be considered coauthors of publications. All other involved persons who also contributed to the study will be listed as the “KAIA-COPD working group”.

## Discussion

This study will investigate whether PA behavior after PR completion can be improved by providing a home-based program via a smartphone application in comparison to usual care after PR and thus addresses two important and long-existing gaps in the context of PR.

First, the study intervention provides an innovative, digital way of providing exercise training in an easy-to-access approach (via smartphone application).

Second, in contrast to most postrehabilitation programs that have been tested so far, the study intervention is focused on increasing or at least maintaining physical activity after completing an in-hospital PR by using the KAIA COPD app and an activity tracker in comparison to usual care (the control group).

Since no standards on placebos for digital interventions exist and the creation of such a control is associated with a high risk of bias, the intervention will be compared to usual care without any specific comparator and with no imposed restrictions on other treatments. Usual care in this context means handing out the German version of the brochure “Living well with COPD” [18], which includes an emergency plan and provides exercise training examples, handing out guidelines from outpatient physiotherapists and handing out a detailed medical report including medical recommendations.

The results of this study might lead to new structured maintenance programs for COPD patients following PR. The findings of this study will be published in peer-reviewed journals and conference presentations.

## Trial status

The trial status is ongoing with the first patient recruited in August 2019. We expect the recruitment to be finished by August 2020 and the last patient last visit to take place in February 2021. The current protocol is version 2 dated 17 December 2019.

## Abbreviations

CAT: COPD Assessment Test
KlinV: Clinical Trial Ordinance
COPD: Chronic obstructive pulmonary disease
CRF: Case report form
eCRF: Electronic case report form
CRQ: Chronic Respiratory Questionnaire
GOLD: Gobal Initiative on Obstructive Pulmonary Disease
FEV1: Forced expiratory volume in one second
FT: Feeling thermometer
FVC: Forced vital capacity
GCP: Good clinical practice
HADS: Hospital Anxiety and Depression Scale
HROoL: Health-related quality of life
PA: Physical activity
PR: Pulmonary rehabilitation
